# An Unusual Ocular Manifestation of Disseminated Tuberculosis: Combined Retinal Vascular Occlusion Inducing Acute Loss of Vision

**DOI:** 10.7759/cureus.62743

**Published:** 2024-06-20

**Authors:** Pradip Kumar Behera, Krishna Padarabinda Tripathy, Debasis Pathi, Sangam Tarun Venkat Mahesh, Manmath K Das

**Affiliations:** 1 General Medicine, Kalinga Institute of Medical Sciences, Bhubaneswar, IND; 2 Ophthalmology, Kalinga Institute of Medical Sciences, Bhubaneswar, IND

**Keywords:** optic nerve infiltration, intra ocular tuberculosis, central retinal artery occlusion (crao), central retinal vein occlusion (crvo), disseminated miliary tuberculosis

## Abstract

Disseminated tuberculosis (TB) is a life-threatening disease caused by the hematogenous spread of Mycobacterium tuberculosis. Acute loss of vision as a symptom of disseminated TB is uncommon, as per the literature. Uveitis is the most common ocular manifestation of TB, and tubercular retinal arterial or venous occlusion, with or without ocular signs, has been rarely described before. We discuss the case of a 34-year-old truck driver who presented with fever, cough, and sudden painless loss of vision in the right eye. Examination revealed optic neuropathy, as well as central retinal artery and venous occlusion. Investigations showed bilateral miliary shadows on chest X-ray and multiple ring-enhancing brain lesions on MRI brain, consistent with disseminated TB. Anti-tubercular therapy led to clinical improvement. We report this case to highlight the rarity of this condition.

## Introduction

In India, tuberculosis (TB) remains a significant public health challenge, despite concerted efforts by governmental bodies such as the Ministry of Health to combat its prevalence. According to the Global TB Report 2022, an estimated 10.6 million cases of TB were reported in 2021. Among the six high-burden countries in the Southeast Asia region, India accounts for 28% of the global TB burden [1]. Disseminated TB is defined as the presence of tubercular lesions at two or more nonadjacent locations due to the hematogenous spread of Mycobacterium tuberculosis. This dissemination can arise from the progressive advancement of primary infection, reactivation of a latent focus followed by dissemination, or, in rare cases, through iatrogenic routes. Disseminated TB is estimated to account for less than 2% of all TB cases and up to 20% of all cases of extrapulmonary TB [2].

Ocular involvement in disseminated TB is a rare entity. Ocular TB can involve any part of the eye and can occur with or without evidence of pulmonary or extrapulmonary TB disease. The most common ocular TB manifestation is granulomatous uveitis. The most typical lesions related to ocular TB are choroidal granulomas, occlusive retinal vasculitis, and multifocal serpiginous-like choroiditis [3]. Central retinal arterial occlusion (CRAO) and central retinal venous occlusion (CRVO) are rare and combined CRAO and CRVO is even rarer [4]. We describe a case of a middle-aged truck driver who presented with disseminated TB (pulmonary, ocular) with CRVO.

## Case presentation

A 34-year-old male truck driver, previously in good health, presented with a fever and a persistent dry cough lasting for one month and sudden, painless loss of vision in the right eye for 10 days. He had no history of similar complaints in the past or any significant medical history. The patient was married and had a history of alcoholism and smoking. On examination, the patient was conscious, well-oriented, febrile, lean-built, with clinical pallor, and stable vitals. Respiratory and cardiovascular examinations were unremarkable. On examination of the eyes, he was unable to perceive light in the right eye with a marked relative afferent pupillary defect. The vision was 6/12 in the left eye. He could only read the first plate of the Ishihara chart with the left eye, suggestive of total color blindness.

A detailed systemic workup was undertaken to investigate the underlying cause. HIV and hepatitis screenings were negative. Chest X-ray in posterior-anterior view showed bilateral miliary shadows. His erythrocyte sedimentation rate (ESR) was 120 mm, and C-reactive protein (CRP) was 131 mg/l. Renal function tests and liver function tests were in the normal range (Table 1). Peripheral smear showed microcytic hypochromic red blood cells, with no parasites. Sputum analysis for gram stain and acid-fast bacilli did not detect any organism. However, CBNAAT of sputum returned positive for Mycobacterium tuberculosis with no resistance to rifampicin.

**Table 1:** Liver function tests, renal function tests, and hemogram SGPT: serum glutamic-pyruvic transaminase; SGOT: serum glutamic-oxaloacetic transaminase; ALP: alkaline phosphatase; GGT: gamma-glutamyl transferase; WBC: white blood cells

Lab parameter	Patient value	Normal range
Serum bilirubin	0.34 mg/dl	0.2-1.2 mg/dl
SGOT	31 U/L	0 - 40 U/L
SGPT	30 U/L	5 - 40 U/L
Serum ALP	81 U/L	40-129 U/L
Serum GGT	70 U/L	10-60 U/L
Serum albumin	2.8 gm/dl	3.5-5.0 gm/dl
Serum urea	12 mg/dl	12-42 mg/dl
Serum creatinine	0.53 mg/dl	0.7-1.3 mg/dl
Serum sodium	130 mmol/L	136-146 mmol/L
Serum potassium	3.6 mmol/L	3.5-5.1 mmol/L
Serum calcium	8.1 mg/dl	8.6-10.3 U/L
Serum uric acid	3.1 mg/dl	3.4-7.0 mg/dl
Hemoglobin	8.1 gm/dl	13-17 gm/dl
WBC count	10,000/μL	4000-10,000/μL
Platelet count	594,000/μL	150,000-410,000/μL

**Figure 1:**
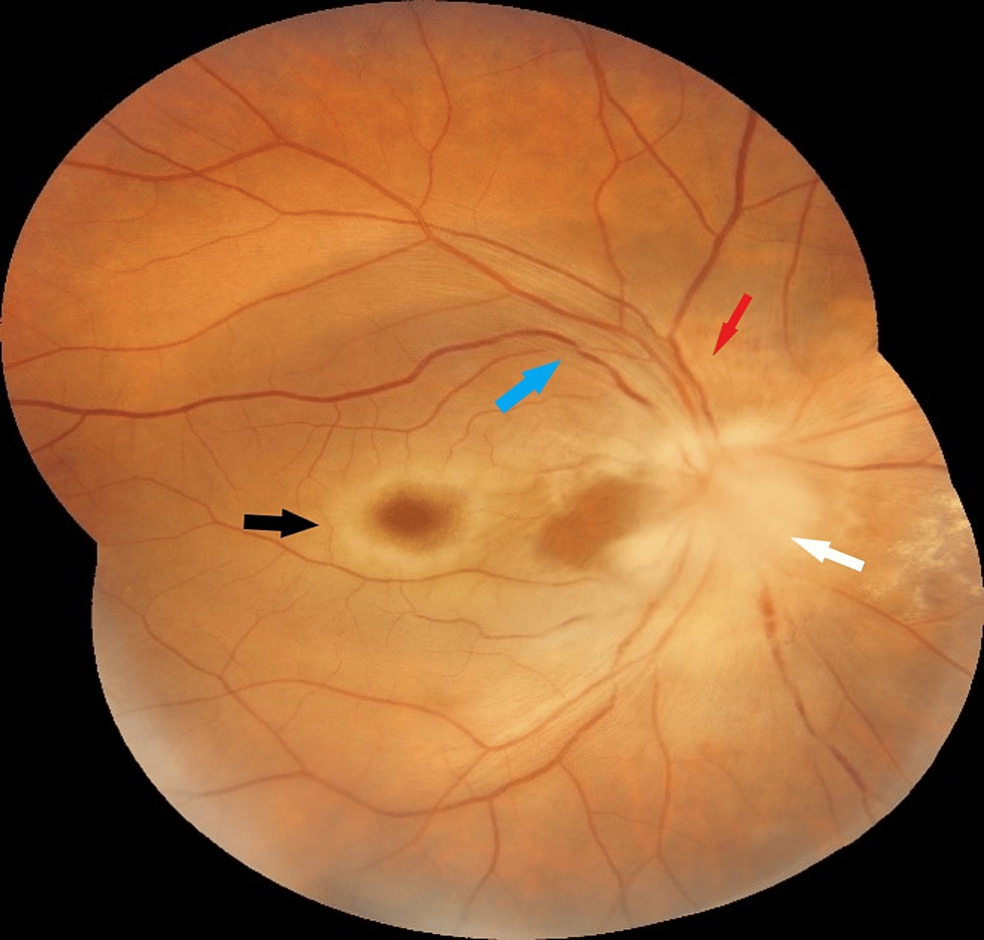
Right eye fundus image White arrow: optic nerve head swelling. Red arrow: infiltration with few flame-shaped hemorrhages in the peripapillary area. Black arrow: a cherry red spot at the macula with a pale retina background. Blue arrow: segmentation of retinal veins

**Figure 2:**
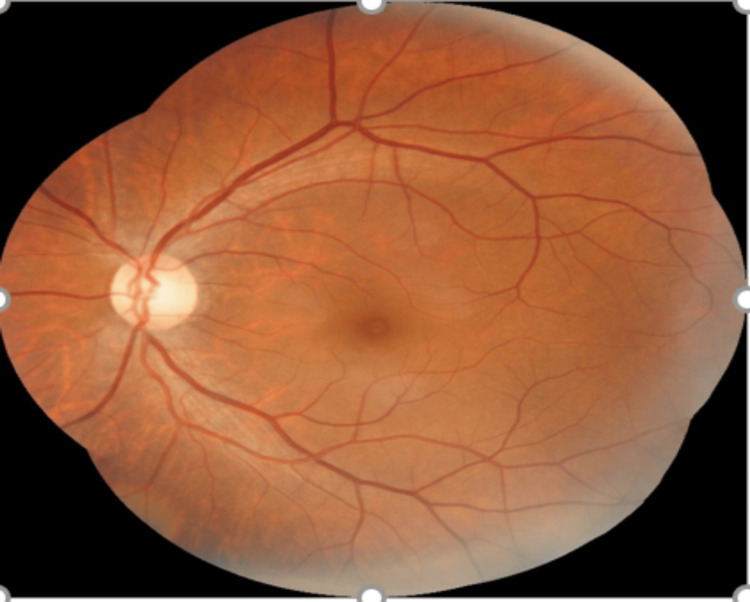
Left eye fundus image

On slit lamp examination, findings were normal in the anterior segment of both eyes. The right eye exhibited a mid-dilated pupil with a relative afferent pupillary defect, while the left eye had a sluggishly reacting pupil. Fundoscopy of the right eye revealed optic nerve head swelling (white arrow) and infiltration with a few flame-shaped hemorrhages (red arrow) in the peripapillary area, along with a cherry-red spot at the macula (black arrow) against a pale retina background. Segmentation of retinal veins (blue arrow) was observed, suggestive of central retinal artery and vein occlusion (Figure 1). On the left eye, fundoscopy showed a normal disc with a cup-to-disc ratio of 0.5:1, while the macula and peripheral retina appeared normal (Figure 2).

**Figure 3:**
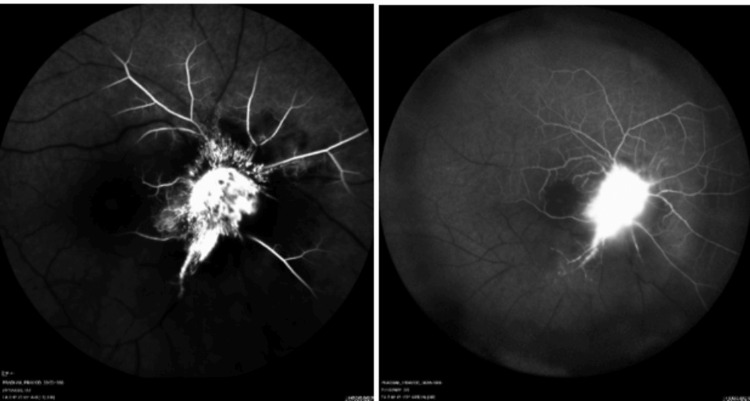
Fundus fluorescein angiography (FFA) of the right eye

**Figure 4:**
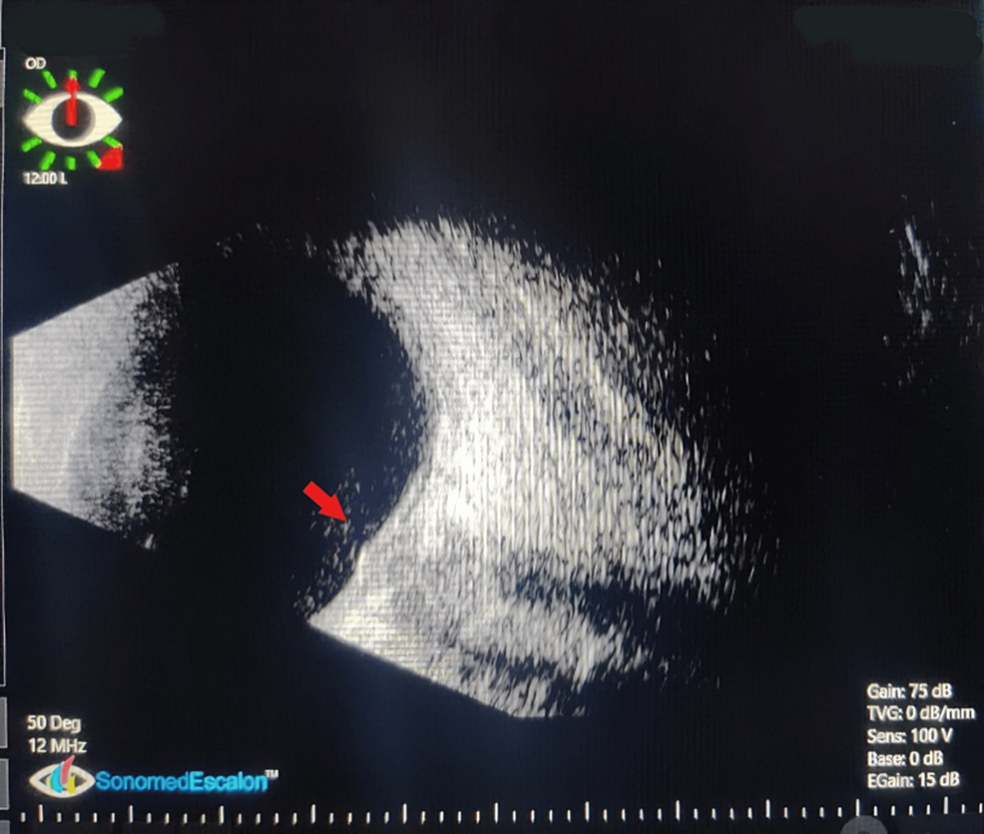
Right eye B-scan Red arrow: raised disc lesion

Fundus fluorescein angiography (FFA) of the right eye revealed an increase in the arm-to-retina time for both the artery (21 seconds) and vein (one minute 13 seconds). Hyper-fluorescence of the optic disc and peripapillary area was evident, accompanied by capillary non-perfusion throughout the rest of the retina, indicative of combined central retinal artery and vein occlusion (Figure 3). Conversely, FFA of the left eye depicted normal dye transit. B-scan analysis of the right eye revealed a raised disc lesion, suggestive of an optic nerve granuloma (Figure 4), while the left eye displayed a normal study.

**Figure 5:**
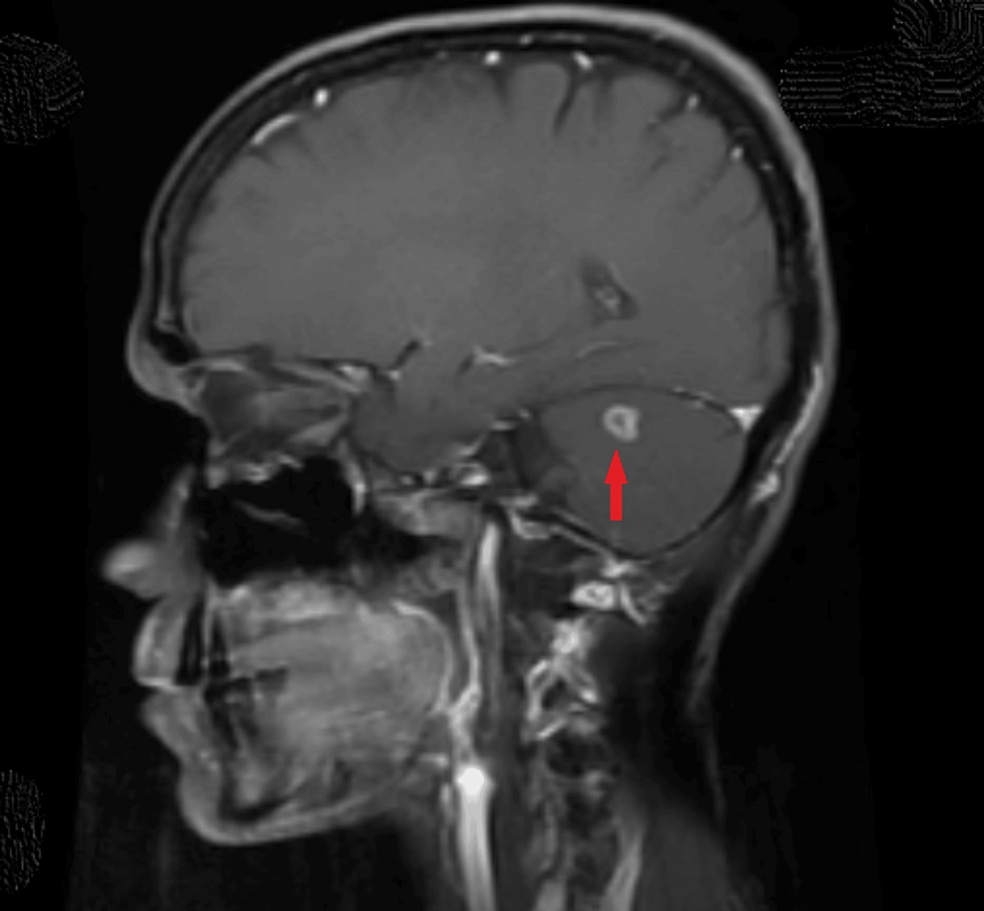
MRI of the brain: sagittal T1 section with contrast Red arrow: well-defined ring-enhancing lesion in the superior part of the right and left cerebellar hemisphere MRI: magnetic resonance imaging

**Figure 6:**
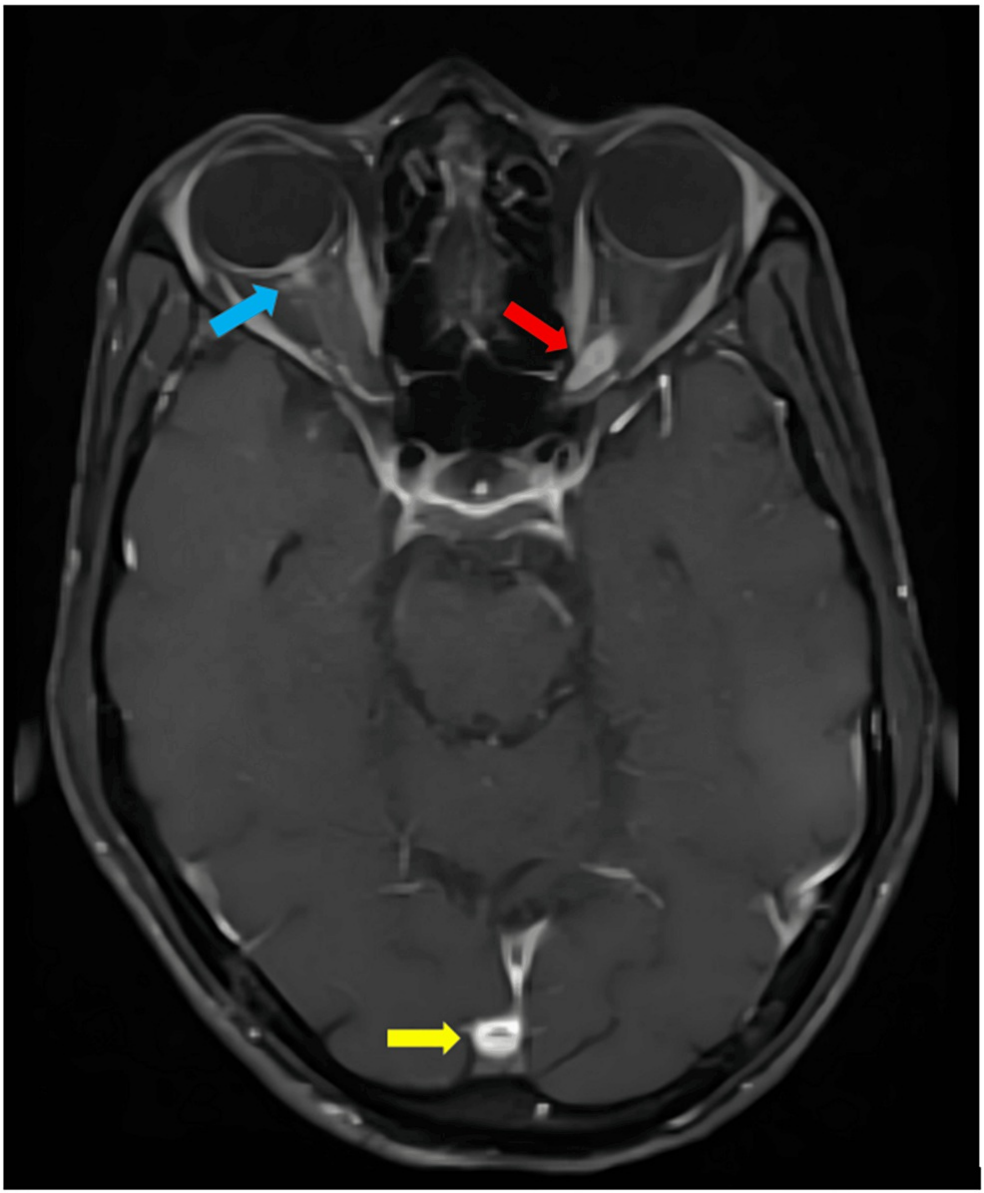
T1W contrast-enhanced MRI orbit Blue arrow: an ill-defined heterogenous enhancing lesion measuring approximately 4 × 2 mm was noted at the optic nerve head causing mild flattening of the posterior sclera; diffuse thickening of the optic nerve in the right optic canal was noted without any extension anterior to the globe. Red arrow: a well-defined heterogenous enhancing lesion measuring approximately 6× 3 mm was noted in the left optic nerve at the apex of the orbit; diffuse thickening with buckling of the optic nerve in the optic canal was noted without any extension beyond the optic canal. Yellow arrow: evidence of a ring-enhancing lesion was noted in the medial right occipital lobe parenchyma almost in the midline MRI: magnetic resonance imaging

**Figure 7:**
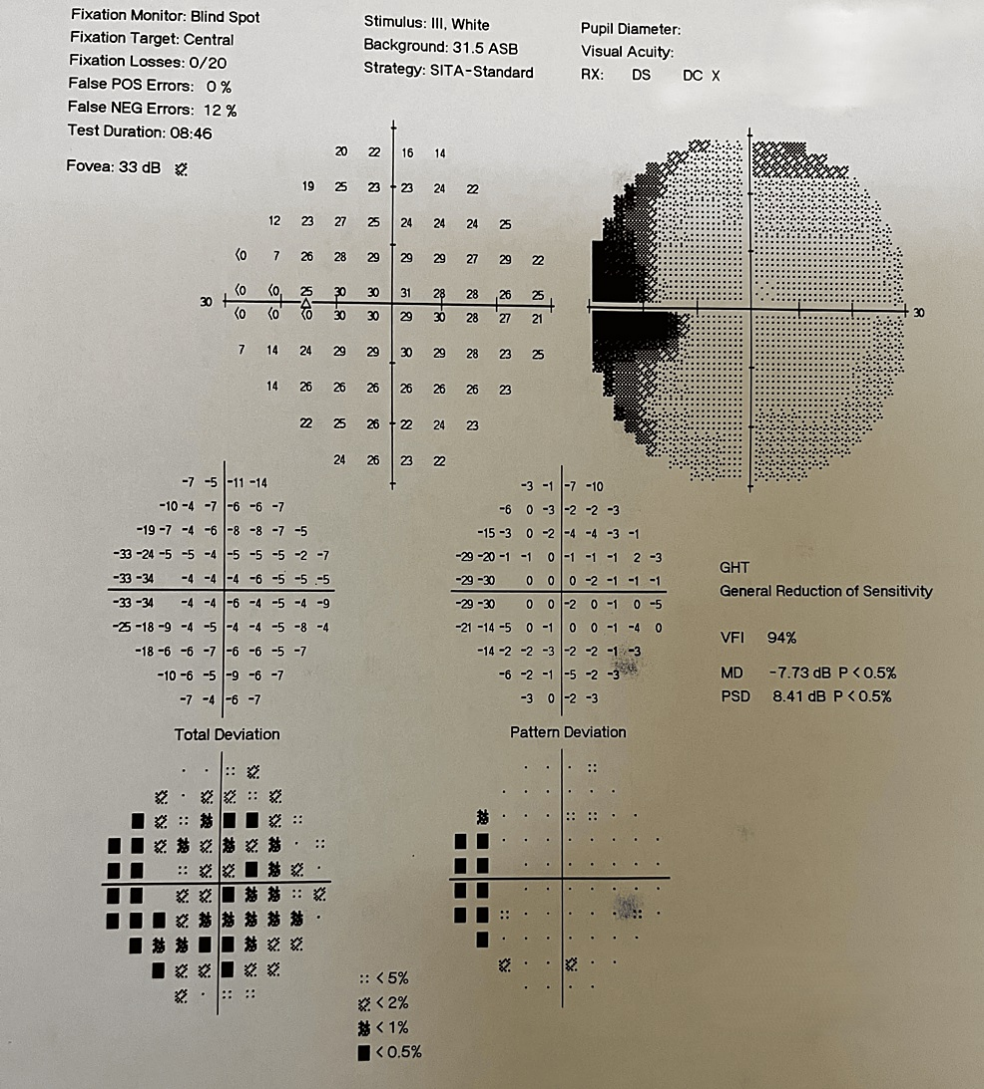
Perimetry of the left eye

HRCT thorax showed a pattern of multiple tiny nodular opacities (tree-in-bud) in bilateral lungs involving all lobes, with a few sub-centimeter lymph nodes seen in the mediastinum - pre tracheal, pre-vascular, and para tracheal regions. MRI brain and orbit with contrast revealed multiple ring-enhancing lesions (seven in number) seen in bilateral parietal and left frontal lobes, as well as bilateral cerebellar hemispheres, with the largest measuring 9.8 x 9.2 mm in the right cerebellar hemisphere (Figure 5). There was an enhancing area in the right optic disc and retro bulbar aspect of the right optic nerve measuring 6 x 4 mm, and a well-defined enhancing lesion measuring 11 x 6 mm seen in the left optic nerve at the posterior orbital apex with an anterior extent seen 15 mm from the optic disc (Figure 6). Perimetry of the left eye was performed given orbital apex involvement by HFA (30-2) and showed incomplete temporal hemianopia (Figure 7).

Anti-tubercular therapy was started according to the patient's weight (46 kg). His condition improved symptomatically after starting anti-tubercular therapy as per the National Tuberculosis Elimination Programme (NTEP), with a dosing of tablet Isoniazid 225 mg (5 mg/kg), tablet rifampicin 450 mg (10 mg/kg), tablet pyrazinamide 1200 mg (25 mg/kg), and tablet ethambutol 825 mg (15 mg/kg) once daily for nine months. The fever subsequently subsided and the patient's condition improved. Liver function tests repeated after seven days were within normal limits.

## Discussion

This report highlights a rare manifestation of disseminated TB involving the ocular system, specifically presenting as optic neuropathy along with central retinal artery and venous occlusion. It underscores the importance of considering TB as a potential etiology in patients presenting with atypical visual symptoms, especially in regions with a high burden of TB like India. Our patient, a 34-year-old truck driver, initially presented with constitutional symptoms such as fever and cough, which are common manifestations of pulmonary TB. However, the sudden painless vision loss in the right eye prompted a thorough ocular examination, which revealed optic neuropathy and central retinal artery and venous occlusion of the right eye. These findings prompted further investigations, ultimately revealing the disseminated nature of the TB infection.

The diagnostic workup revealed several key findings consistent with disseminated TB. Chest X-ray findings of bilateral miliary shadows, along with positive sputum analysis for Mycobacterium tuberculosis, confirmed the pulmonary involvement. Additionally, MRI findings of multiple ring-enhancing brain lesions further supported the diagnosis of disseminated TB with central nervous system (CNS) involvement. Ocular TB, although less common than pulmonary TB, can manifest in various forms, including choroidal granulomas, occlusive retinal vasculitis, and multifocal serpiginous-like choroiditis [3]. Our patient presented with optic neuropathy and central retinal artery and venous occlusion, which are rare ocular manifestations of TB but have been reported in the literature [5].

The initiation of anti-tubercular therapy led to clinical improvement, as evidenced by the resolution of constitutional symptoms and improvement in ocular findings. This underscores the importance of early diagnosis and prompt initiation of treatment in cases of disseminated TB to prevent further complications and mitigate morbidity. Furthermore, this case report emphasizes the need for a comprehensive approach to the diagnosis and management of TB, particularly in patients with atypical presentations. In regions with a high TB burden, healthcare providers should maintain a high index of suspicion for TB, even in cases presenting with uncommon manifestations such as ocular involvement.

## Conclusions

This case report underscores the importance of considering disseminated TB in patients presenting with atypical visual symptoms and systemic manifestations, particularly in regions with a high burden of TB like India. Early recognition, diagnosis, and initiation of anti-tubercular therapy are crucial for improving patient outcomes and preventing further complications associated with disseminated TB.
